# Commentary: The Relationship between Sleep Complaints, Depression, and Executive Functions on Older Adults

**DOI:** 10.3389/fpsyg.2016.01870

**Published:** 2016-11-28

**Authors:** Andrea Ballesio, Caterina Lombardo

**Affiliations:** Department of Psychology, La Sapienza University of RomeRome, Italy

**Keywords:** sleep, insomnia, depression, frontal lobe functions, executive functions, commentary

Executive functions are a set of top-down higher order cognitive processes that are vulnerable to sleep loss (Gorgoni et al., 2014) and impaired in individuals with depression (Snyder et al., 2015). The co-occurrence of impaired executive functions, sleep disturbance and depressive symptoms in elderly samples indicates that the three conditions may be interrelated (Yu et al., 2016). Nevertheless, to date few studies investigated the relationships between these three variables. The present commentary aims to draw attention to some factors that may be considered for advancing knowledge in this field, starting from findings reported by de Almondes et al. (2016) in the article recently published in “Frontiers in Psychology.”

In that paper, de Almondes et al. (2016) examined older adults without major medical or psychiatric illnesses, who underwent a neuropsychological assessment including tasks measuring core executive functions of inhibitory control, cognitive flexibility, and working memory. Presence of depressive symptoms and sleep complaints was also assessed through self-reported measures. Findings from regression analyses evidenced that both sleep complaints and depression were associated with poor performance in executive functions tasks. Results appear promising, although some factors may be considered to improve future research in the field.

First, the authors refer to sleep complaints measured through a self-report item asking participants whether they suffered from “sleep related problems in recent months or weeks.” However, this approach may encompass some limitations. First, “sleep complaints” is an umbrella term that refers to different types of clinical or subclinical conditions such as insomnia, presence of non-restorative sleep or fragmented sleep, parasomnias, sleep-related breathing disorders, circadian rhythm disturbances, etc. “Sleep complaints” may also refer to difficulties related to voluntary sleep deprivation (i.e., due to life style factors). Importantly, all these conditions may have different effects on cognitive functions. For instance, Shekleton et al. (2010) suggested that the presence of insomnia disorder may reduce accuracy and increase the number of errors in performance tasks, while sleep deprivation may result in longer reaction times. Thus, a clearer definition of sleep complaints is needed to advance knowledge in the field. Second, the lack of validity and reliability of the measure used to assess sleep quality may have further weakened the results. Polysomnograpy (PSG) is considered the gold-standard for the objective assessment of sleep quality and sleep disorders other than insomnia (Riemann et al., 2015). Self-report questionnaires are also available and widely used for measuring sleep quality (Buysse et al., 1989) and insomnia severity (Bastien et al., 2001). Thus, future studies in the field are strongly encouraged to include valid and reliable measures of sleep quality.

The inclusion of standardized measures of sleep disturbance and symptoms severity would additionally allow to explore the reciprocal relationships between poor sleep, executive functions and depression. As aforementioned, executive functions are impaired in major depression (Snyder et al., 2015), and are vulnerable to poor sleep (Shekleton et al., 2010). Within this frame, depressive symptoms may moderate the relationship between poor sleep and impaired executive functions, as previously suggested (Yu et al., 2016). Specifically, when present, depressive symptoms may increase the impact of poor sleep on executive functions. Nevertheless, poor sleep is also a predictor of major depression (Baglioni et al., 2011). Thus, it is also plausible to hypothesize that poor executive functions may mediate the relationship between poor sleep and depression. That is, poor sleep may impair executive functions, exposing individuals to use maladaptive regulatory strategies associated with poor executive functions such as rumination, worry, difficulties in emotion regulation, which may lead to depression. Nevertheless, to the best of our knowledge no study have tested this hypothesis and thus future studies are needed.

To conclude, the study of de Almondes et al. (2016) highlight the presence of an association between poor sleep, depressive symptoms and executive functioning in older adults. Results appear promising, although replication studies are needed that address also the issue of the reciprocal influence between sleep quality, depressive symptoms severity and executive functioning using a clearer definition of sleep complaints and valid and reliable instruments for their assessment.

## Author contributions

AB contributed writing the draft of the manuscript; CL contributed revising the paper.

### Conflict of interest statement

The authors declare that the research was conducted in the absence of any commercial or financial relationships that could be construed as a potential conflict of interest.
